# Basal level of FANCD2 monoubiquitination is required for the maintenance of a sufficient number of licensed-replication origins to fire at a normal rate

**DOI:** 10.18632/oncotarget.1796

**Published:** 2014-03-13

**Authors:** Jayabal Panneerselvam, Anna Pickering, Bing Han, Liantao Li, Junnian Zheng, Jun Zhang, Yanbin Zhang, Peiwen Fei

**Affiliations:** ^1^ University of Hawaii Cancer Center, University of Hawaii, Honolulu, HI; ^2^ Jiangsu Key Laboratory of Biological Cancer Therapy, Xuzhou Medical College, China; ^3^ Department of Laboratory Medicine and Pathology, Mayo Clinic, Rochester, MN; ^4^ Department of Biochemistry and Molecular Biology, University of Miami Miller School of Medicine, Miami, FL

**Keywords:** Replication, FANCD2

## Abstract

Normal DNA replication starts following the stepwise recruitment of replication initiators to assemble Mini-chromosome Maintenance (MCM) 2-7 protein complexes at an adequate amount of DNA replication origins. Under normal conditions, the monoubiquitination of Fanconi Anemia (FA) group D2 protein (FANCD2) occurs in each S-phase of cell cycle, which is the basal level of FANCD2 monoubiquitination. However, little is known regarding the roles of this basal level of monoubiquitinated FANCD2. Here we show that monoubiquitinated FANCD2 in each S-phase of normal cell cycle is essential for replication origins to fire at a normal rate. We found that the basal level of the monoubiquitinated FANCD2 can interact with replication origins as well as mini-chromosome maintenance protein 3 (MCM3) in an S-phase specific manner to secure an enough number of the licensed-origins to fire. Non-monoubiquitinated FANCD2 or mutant MCM3 lacking AA 477-480 responsible for interacting with FANCD2 can lead to an insufficient amount of licensed origins to fire and, thereby, enlarged intervals between the fired origins. Our results demonstrate that the monoubiquitinated FANCD2 in each S-phase of normal cell cycle is required to maintain an enough number of licensed origins to initiate the normal DNA replication. This finding is the first to provide insights into how FANCD2 functions under normal condition of cell cycle to maintain genome stability, as well as resulting implications in the strategic improvement for the fight against human cancer.

## INTRODUCTION

DNA replication licensing occurs from late M phase to G1 phase to ensure that DNA is replicated in the coming S-phase only once per cell cycle [1-5]. Within the licensing process, the ORC (origin recognition complex) is bound to replication origins throughout the cell cycle, providing a platform for Cdc6 and Cdt1 in G1 to recruit MCM (Mini-chromosome maintenance) proteins, which unwind DNA to fire DNA replication in S-phase [6-8]. Before the beginning of DNA replication or origin firing, an adequate amount of MCM-assembled/licensed origins is needed to have a normal rate of origin firing. Deregulation of MCM function, thus interfering with the origin firing, has been linked to genomic instability and a variety of carcinomas [6, 9, 10].

Fanconi Anemia (FA) group D2 protein (FANCD2) is activated through monoubiquitination at K561 during DNA replication or upon DNA damage [11-14]. Much effort has been made to understand the roles that FANCD2 plays upon DNA damage [11, 13, 15-19] and in the subsequent suppression of the development of human cancer [20-23]. However, little is known regarding the roles of the normally monoubiquitinated FANCD2 in DNA replication, within which the entirety of cellular DNA must be faithfully duplicated to maintain genome stability and protect cells from neoplastic transformation. We report that in normally cycling cells, the basal level of FANCD2 monoubiquitination is required to work with replication initiator(s) for the maintenance of an adequate amount of licensed origins to fire. This finding is the first to provide insights into how FANCD2 acts under normal condition of cell cycle to maintain genome stability, advancing our understanding how Fanconi Anemia tumor suppressor signaling pathway functions under normal condition besides conditions of stress.

## RESULTS AND DISCUSSION

### Monoubiquitinated FANCD2 Strongly Interacts with Replication Origins *in vivo* and *in vitro*

**Figure 1:**
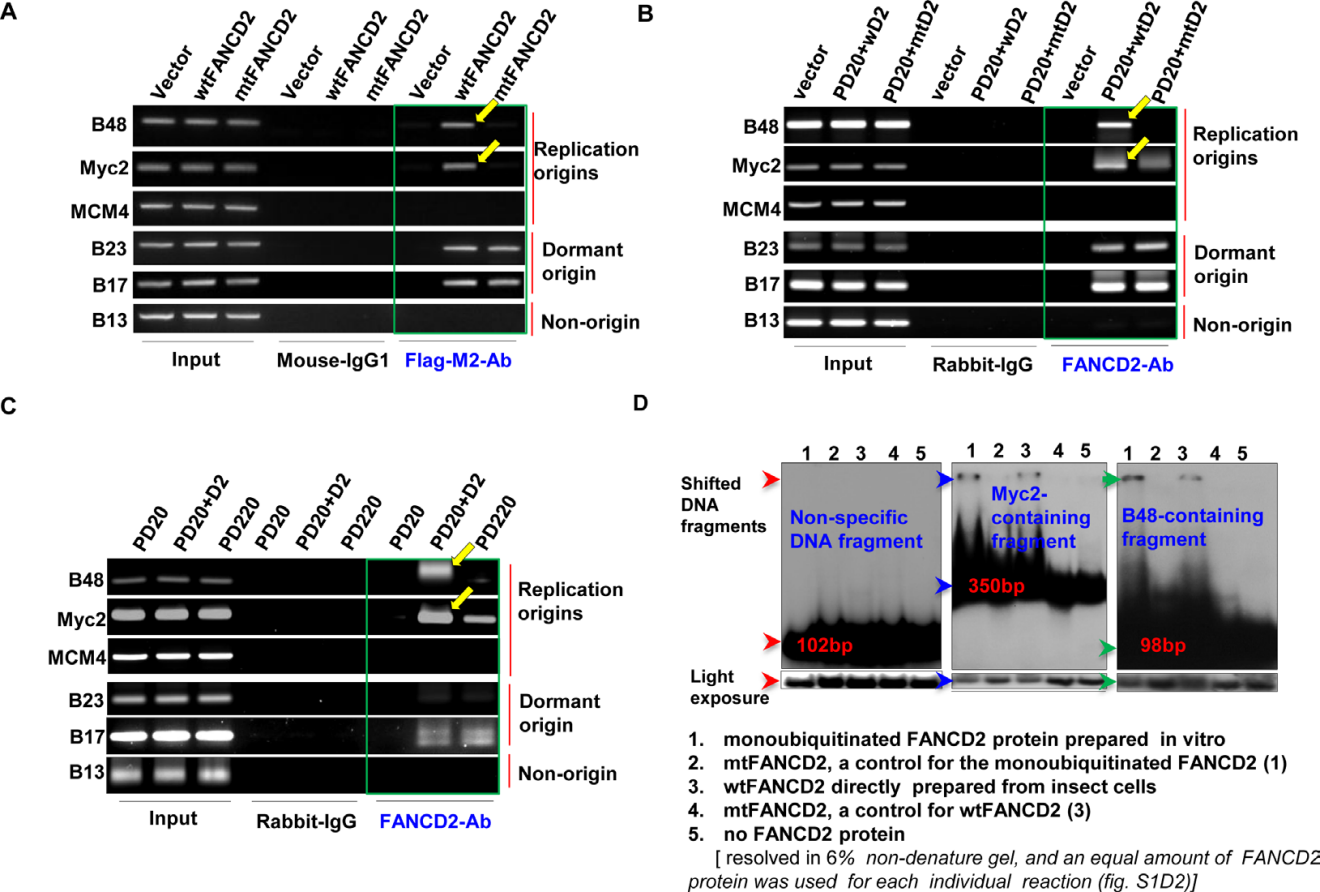
Monoubiquitinated FANCD2 strongly interacts with replication origins in vivo and in vitro Flag-wtFANCD2, but not Flag-mtFANCD2, strongly interacts with B48 and Myc2 replication origins in normally growing HEK293 cells. HEK293 cells were transiently transfected with empty vector, Flag-wtFANCD2 and Flag-mtFANCD2. 48 hr post-transfection, cells were fixed with 1% paraformaldehyde for ChIP assay. ChIP PCR primers for five origins were designed upon published sequences. As yellow arrowheads pointed, Flag antibodies pulled down a low amount of origin B48 or Myc2 -containing DNA fragments from the lysates of cells transfected with Flag-mtFANCD2, as compared to the cells transfected with Flag-wtFANCD2. Plasmids used were equally transfected into cells (Supplementary Fig. 1A2-4). Mouse-IgG1 was used as IP control and did not pull down any noticeable amount of DNA fragment. 5% of the IP-lysate was used to prepare PCR template as an input control. B. WtFANCD2, but not K561R mtFANCD2, clearly interacts with the replication origins in FA derivative cells. PD20 FA cells (FANCD2-/-) were used to establish stably-transfected cells carrying wtFANCD2 or mtFANCD2/inactivated FANCD2 with a similar cell proliferation profile with an exception of 2-3% less in S-phase population upon cells carry inactivated FANCD2 (Supplementary Fig. 1C1, c2). ChIP assays on these FA derivative cells [expressing a comparable level of FANCD2 protein (Supplementary Fig. 1C3)] were performed by using FANCD2 antibodies, which can pull down more B48 and Myc2 origin-containing DNA fragments from the lysates of cells carrying wtFANCD2, as compared to cells harboring mtFANCD2 (D2 labeled in the figure means FANCD2). C. FANCD2 is insufficiently associated with B48 and Myc2 origins in PD220 FA cells (FANCA-/-). FANCD2 ChIP assays on PD20, PD20+wtFANCD2, or PD220 cells were conducted. Equal amount of FANCD2 protein levels was confirmed in PD20+wtFACD2 cells and PD220 cells (Supplementary Fig. 1C3). FANCD2 antibodies can pull down more B48 and Myc2 origin-containing DNA fragments in PD20+wtFANCD2 cells not in PD220 cells (as a result of FANCA-/-, FANCD2 cannot be monoubiquitinated). D. Myc2 and B48 origin-containing DNA fragments strongly bind to monoubiquitinated FANCD2, but not un-monoubiquitinated or mtFANCD2. Ubiquitin-conjugated or –unconjugated FANCD2 and mtFANCD2 proteins (Supplementary Fig. 1D) were incubated with 32p-labeled DNA segments containing B48, Myc2, MCM4, B23, B17 or B13. The resulting complexes were analyzed on a 6% polyacrylamide non-denature gel. As pointed by blue and green arrowheads, B48 and Myc2 strongly bind to monoubiquitinated FANCD2 among all forms of FANCD2 proteins tested, indicated by the shifted 32p-labled DNA segments. Like non-origin fragment B13 (shown), B23 and B17, as well as MCM4 appeared to be incapable of binding well to any forms of FANCD2 proteins under the assay condition here used (not shown).

Given a little known role of the normally monoubiquitinated FANCD2 in DNA replication, we asked whether FANCD2 has any effects on the very beginning of the replication process: the normal replication origin firing that pre-requires an adequate number of replication origins assembled ready with MCM complexes [10, 24-27]. First, we asked whether FANCD2 is localized at origins of DNA replication, noting that monoubiquitinated FANCD2 interacts with chromatin [28]. We thus examined if FANCD2 can associate with replication origins: MCM4, c-Myc (Myc2), Lamin B2 (B48) and GM-CSF (B17 & B23) (Supplementary Fig. 1A1) [29]. To date, the investigation into replication origins was mostly derived from these few well-known origins [29-31] because other origins, functioning in each S-phase of normal cell cycle, are poorly characterized [32]. Surprisingly, using chromatin immunoprecipitation (ChIP) we found that Flag-wild type (wt) FANCD2 can pull down more B48 or Myc-2 origin-containing DNA fragments, as compared to Flag-mutant (mt) FANCD2 K561R (K561R-losing the capacity to be monoubiquitinated/activated during S-phase of cell cycle or upon DNA damage) (Fig. 1A and Supplementary Fig. 1A2-4). We also performed similar ChIP assay using the same cells transfected with Flag-wtFANCI or mtFANCI K523R (similarly, the mutant losing the capacity to be monoubiquitinated/activated) given the fact that FANCD2 and FANCI are known to function in the same protein complex, named “ID complex” [33]. We found that wtFANCI, unlike wtFANCD2, cannot pull down more origin-containing DNA fragments in comparison with mtFANCI under the same condition applied for Flag-FANCD2 ChIP (Supplementary Fig. 1B), suggesting wtFANCI does not play the same function as does wtFANCD2 under the conditions studied. The capability of wtFANCD2, but not mtFANCD2, to interact with origin DNA was further validated in normally growing FA cells, including stably-transfected PD20 cells (FANCD2-/-) complemented with wt or mtFANCD2 and PD220 cells (FANCA-/-) (Fig. 1B, C and Supplementary Fig. 1C). Collectively, in normally growing cells, wtFANCD2 is involved in DNA replication initiation via its association with DNA replication origins, which is not involving the “ID” complex. To learn more about this association between FANCD2 and replication origins, we performed *in vitro* binding assays using monoubiquitinated FANCD2 (equivalent to wtFANCD2 in S-phase), un-monoubiquitinated FANCD2, or mtFANCD2 derived from a baculovirus-expression system, coupled with *in vitro* FANCD2 monoubiquitination[19]. As shown in Figure 1D and Supplementary Figure 1D, monoubiquitinated FANCD2 can clearly shift more origin-containing DNA fragments as compared to un-monoubiquitinated FANCD2 and mtFANCD2. Together, these results demonstrate that normally monoubiquitinated FANCD2 participates in replication initiation possibly through a direct interaction with replication origins. To date, it is unclear whether there is a consensus or loosely related sequence conserved in replication origins, which would permit a relatively high binding affinity for monoubiquitinated FANCD2 observed above. This binding preference, we think, might result from “a better structural fit” formed between replication origins and monoubiquitinated FANCD2 in normally growing cells on the basis of the fact that ChIP and *in vitro* binding assays support each other (Fig.1 and Supplementary Fig. 1).

### Cells Carrying a Low Basal Level of FANCD2 Monoubiquitination Show a Low Amount of Licensed Origins to Fire

**Figure 2:**
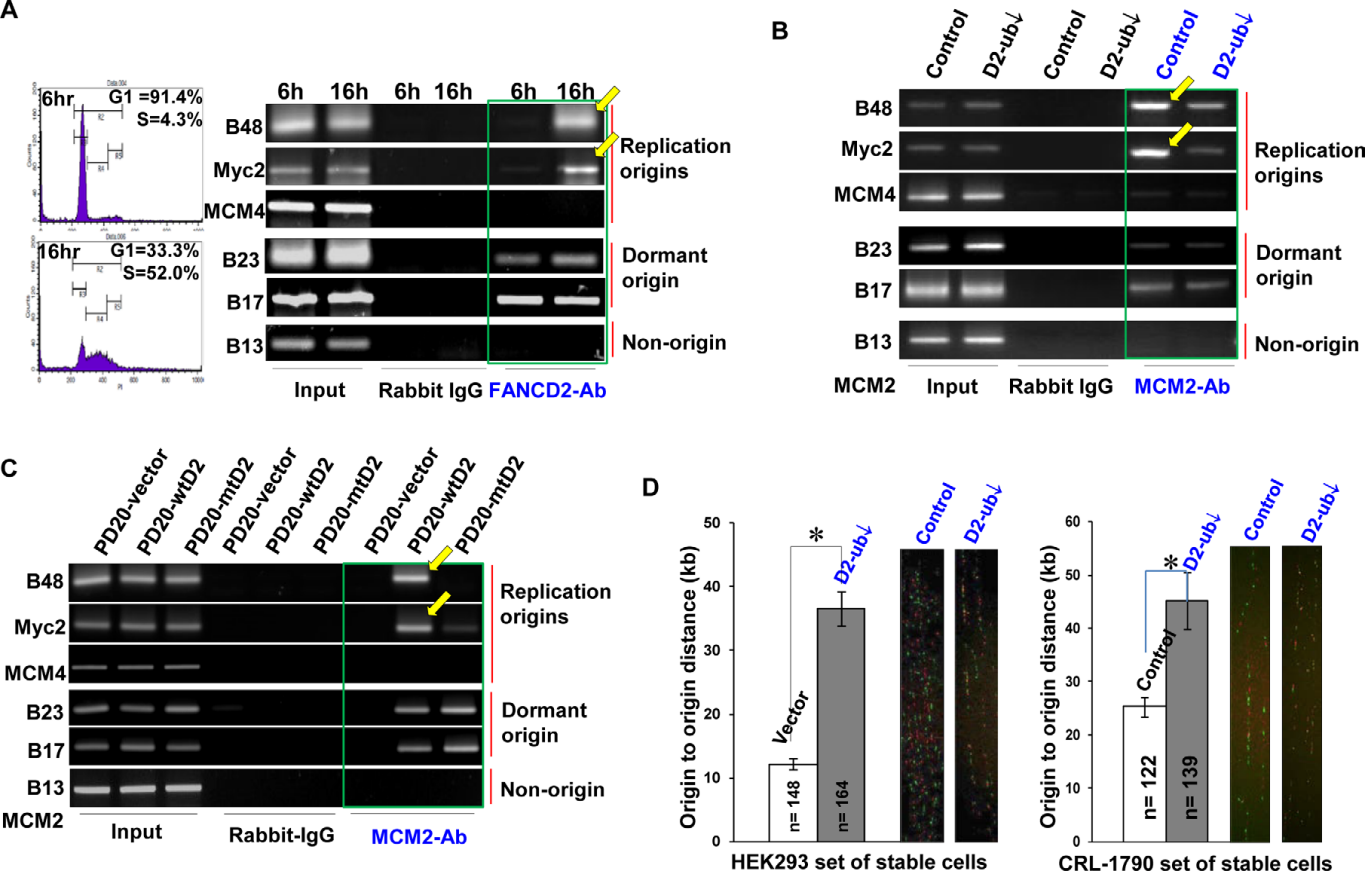
Cells Carrying a Low Basal Level of FANCD2 Monoubiquitination Show a low Amount of Origin DNA associated with MCM2 and a Decreased Rate of Origin-firing A. FANCD2 associates with replication origins in an S-phase specific manner. Using FANCD2 antibodies along with rabbit IgG antibody control, we performed ChIP analyses on UV treated as well as starvation-synchronized CRL-1790 cells cultured in regular medium for 6, 12, 14 and 16 hrs, respectively. FANCD2 antibodies can pull down much more B48 and Myc2 origin-containing DNA fragments from the lysates of the pool cells cultured in regular medium for 16 hr and harboring 52% of S-phase cells, as compared to the other pools of cells carrying a lower percentage of S-phase cells. In non-stressed cells, the level of FANCD2 monoubiquitination appears to be positively associated with the amount of pulled-down origin-containing DNA fragments (See FACS data and the basal level of FANCD2 monoubiquitination in Supplementary Fig. 2A1). B. There is a low amount of licensed-origins (origins associated with MCM2) in HEK293 or CRL-1790 cells carrying a reduced basal level of FANCD2 monoubiquitination, as compared to the corresponding control cells carrying a normal basal level of FANCD2 monoubiquitination. Both sets of HEK293 and CRL-1790 stable cells isogenic to FANCL expression (leading to a low basal level of FANCD2 monoubiquitination) were established, which provide two similar systems carrying a normal or reduced basal level of FANCD2 monoubiquitination (Supplementary Fig. 2B1). ChIP assays on these two sets of normally growing CRL-1790 and HEK293 cells were performed. MCM2 antibodies, but not ORC antibodies (Supplementary Fig. 2B2- ORC ChIP assay on CRL-1790 cells; Supplementary Fig. 2B3 – MCM2 and ORC ChIP assays on HEK293 cells), pulled down a low amount of B48 and Myc2-containing DNA fragments in cells carrying a reduced basal level of FANCD2 monoubiquitination, as compared to corresponding control cells containing a normal basal level of FANCD2 monoubiquitination. C. A low amount of origins are associated with MCM2 in FA cells carrying mt or inactivated FANCD2 in comparison with FA cells carrying wtFANCD2. MCM2 ChIP assay on FA derivative cells was performed. These FA derivative cells are PD20 cells (FANCD2-/-) complemented with wtFANCD2, in comparison with mtFANCD2 (K561R); PD220 cells (FANCA-/-, resulting in inactivated FANCD2). MCM2 antibodies only can clearly pull down B48 or Myc2 origin-containing DNA fragments in FA cells carrying wtFANCD2, but not FA cells carrying mt or inactivated FANCD2 (Supplementary Fig. 2B4). D. The origin-firing rate is decreased in normally growing cells carrying a compromised basal level of FANCD2 monoubiquitination. DNA fiber assay was conducted by using CRL-1790 or HEK293 set of stable cells. The distance between fired origins was enhanced in the cells carrying a compromised basal level of FANCD2 monoubiquitination as compared to the corresponding control cells carrying a normal basal level of FANCD2 monoubiquitination. In cells carrying a reduced basal level of FANCD2-monoubiquitination, the number of fired-origins was significantly low as compared to the corresponding control cells (T test, p<0.01). Bars show standard error of the mean (SEM). The images of labeled DNA fibers are shown accordingly at right.

To directly emphasize the specific role of the basal level of FANCD2 monoubiquitination or the normally monoubiquitinated FANCD2 in each S-phase of cell cycle (Fig. 1), we performed FANCD2 ChIP assay using synchronized cells enriched in G1 (G1=91.4%) or S (S=52.0%) phase, noting that FANCD2 gets monoubiquitinated in S-phase, but not in G1 phase, in normally growing cells [11]. We found that endogenous FANCD2 associated well with origins in a manner of depending on the percentage of S-phase cells or the level of monoubiquitinated FANCD2 (Fig. 2A and Supplementary Fig. 2A). We also found that the amount of origin-containing DNA fragments pulled down by FANCD2 antibodies is positively associated with the levels of FANCD2 monoubiquitination in cells under only normal condition but not the condition of stress (Fig. 2A, UV-treated). Apparently, it is the normally monoubiquitinated FANCD2 in unstressed cells that plays substantial roles in the interaction with replication origins. The beginning of a normal S-phase requires an adequate amount of origins pre-assembled with MCM complexes (licensed-origins). We thus asked whether the basal level of FANCD2 monoubiquitination (S-phase only) influences the quantity of licensed-origins (S-phase) in normally growing cells. We generated stable cell pairs (Supplementary Fig. 2B1) isogenic to the basal level of monoubiquitinated FANCD2 using HEK 293 cells (CRL-1573) and colon epithelial cells (CRL-1790), both of which possess stable genetic background suitable for studying basic cellular processes (e.g. replication initiation), as opposed to tumor cells. Using ChIP assays we found that the amount of origin-containing DNA fragments associated with MCM2 (representing MCM complex) is declined in cells carrying a reduced basal level of monoubiquitinated FANCD2, as compared to corresponding control cells carrying a normal basal level of monoubiquitinated FANCD2 (Fig. 2B and Supplementary Fig. 2B2, B3). This indicates that the quantity of licensed-origins (MCM assembled-origins) is decreased in the basal level of FANCD2 monoubiquitination-compromised cells. In contrast, ORC appears to be associated with a similar amount of origin-containing DNA fragments, regardless of the basal level of FANCD2 monoubiquitination (Supplementary Fig. 2B2, B3). In addition, when performing MCM2 ChIP using FA cells carrying monoubiquitinated or un-monoubiquitinated FANCD2 (Supplementary Fig. 1C), we found that MCM2 antibodies remained to pulldown a low amount of replication origins in FA cells carrying either endogenous or exogenous un-monoubiquitinated FANCD2 (Fig. 2B and Supplementary Fig. 2B4). We also noticed that FANCL-downregulation leads to a pool of normally growing cells carrying a low percentage of cells in S-phase (Supplementary Fig. 2C1). We adjusted ChIP input to have an equal amount of S-phase chromatin and found that MCM2 antibodies still pulled down a reduced amount of origin-containing DNA fragments in cells carrying a reduced level of FANCD2 monoubiquitination as compared to cells harboring a normal basal level of monoubiquitinated FANCD2 (Supplementary Fig. 2C2). Therefore, the number of S-phase cells cannot account for a significant drop in the amount of origin-containing DNA segments associated with MCM complex. This observation is also not a result of down-regulated FANCL that would have affected MCM2-7 protein expression (Supplementary Fig. 2C3). In addition, these results were further supported by a low amount of MCM2 proteins at the chromatin level (Supplementary Fig. 2D1, D2). Collectively, the basal level of FANCD2 monoubiquitination (S-phase-specific) can maintain a proper amount of licensed-replication origins. Next we hypothesized that cells harboring un-monoubiquitinated FANCD2 and, thus, carrying an insufficient number of licensed-origins (Fig. 2A-C), may exhibit an abnormal origin firing. To this end, we examined the effect of a deficiency in the basal level of monoubiquitinated FANCD2 on replication-origin firing. As shown in Figure 2D, there is a significant increase in the distances between fired origins in cells carrying a reduced basal level of monoubiquitinated FANCD2 as compared to cells carrying a normal basal level of monoubiquitinated FANCD2. This indicates that the number of fired origins is decreased when S-phase monoubiquitination of FANCD2 is deficient. This further rules out a possibility that the reduced amount of origin-DNA associated with MCM2/MCM2-7 might result from slowly growing cells (FANCL-silenced), which would mislead to a false positive MCM2-ChIP result. Because, the enlarged intervals between fired origins in cells carrying a reduced basal level of FANCD2 monoubiquitination comparing to a normal basal level of FANCD2 monoubiquitination are represented *in situ* by DNA fiber assay, which *in vivo* supports the conclusion that the normal basal level of FANCD2 monoubiquitination is required for replication origins to fire at a normal rate.

### Normally Monoubiquitnated FANCD2 Interacts with MCM3 to Maintain an Optimal Size of MCM2-7 -containing functional complex

**Figure 3:**
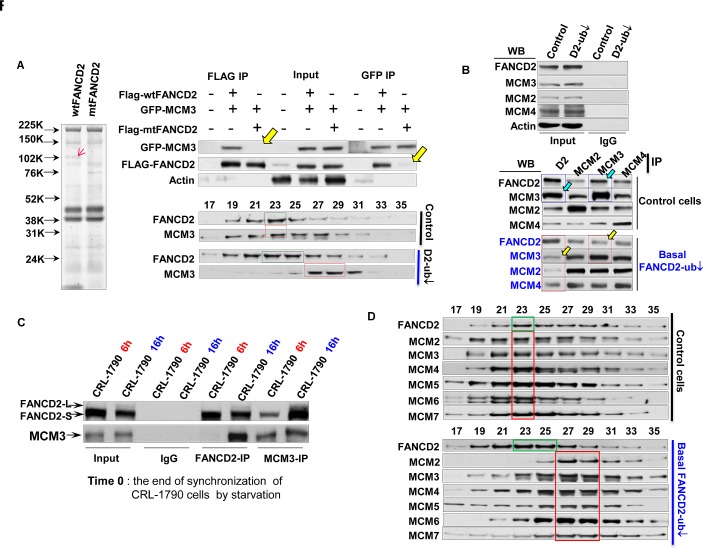
Normally Monoubiquitinated FANCD2 Interacts with MCM3 in an S-phase specific manner and Maintains the Optimal Function of MCM2-7-Containing Protein Complex A. FANCD2 interacts with MCM3. Left panel: Simple blue-staining (taken in gray color) of an 8-16% gradient gel shows “an extra band” in one lane and not in the other control lane. The gel was resolved with Flag-IP elutes, which were prepared from Flag-wtFANCD2 or mtFANCD2 transfected CRL-1790 cells. “The extra band” pointed by a red arrowhead was sliced for mass spectrometry (The equal transfection efficiency of CRL-1790 cells with Flag-wt or mt FANCD2 is shown in Supplementary Fig. 3A). Right panel: CRL-1790 cells were used for transient co-transfection of GFP-fused MCM3 and Flag-wtFANCD2 or Flag-mtFANCD2 K561R. Cell lysate were prepared 48 hr post-transfection. Both Flag and GFP antibodies were used to perform reverse immunoprecipitation (IP) and Western blotting (WB). As yellow arrowheads indicated, Flag-mtFANCD2, unlike Flag-wtFANCD2, is not clearly associated with GFP-MCM3 (top part) (The transfection efficiency was comparable among transfected cells, please see IP input). The sepharose 6B chromatography (gel filtration) was performed using normally growing CRL-1790 set of stable cells (Supplementary Fig. 2B1 left panel). The endogenous FANCD2 co-peaks with endogenous MCM3 in cells carrying a normal basal level of FANCD2 monoubiquitination (middle part), but not in the cells carrying a compromised basal level of FNACD2 monoubiquitination (bottom part) (Fractions #9 and #30 correspond to the size markers of 2000 kDa and 669 kDa respectively, and the whole panel of markers is shown in Supplementary Fig. 3C). B. Confirmation of the interaction between endogenous MCM3 and FANCD2. Pool fractions from #21 to #25 were used to perform reverse IP-WB between FANCD2 and MCM3, MCM2 or MCM7, which also are a representative for MCM5, MCM6 or MCM4, respectively, given three sub-complexes (MCM3&5, MCM2&4, and MCM7&4) formed among MCM family members. FANCD2 appears to interact primarily with MCM3, but not with MCM2 or MCM7, whose antibodies pulled down a low amount of FANCD2 protein as compared to MCM3 antibodies. Vice versa, FANCD2 antibodies pulled down more MCM3 proteins as compared to the amount of MCM2 or MCM7 proteins pulled down. C. The Interaction between MCM3 and FANCD2 proteins is S-phase Specific. The reverse IP-WB of MCM3 and FANCD2 was performed using the same batch of synchronized cells used (Fig. 2A). The FANCD2 antibodies can pull down more MCM3 proteins from the lysates of the cell pool enriched in S but not in G1 phase of normal cell cycle. Similarly, MCM3 antibodies can pull down more FANCD2 proteins from the lysates of the cell pool enriched in S-phase, but not in G1 phase. D. The Size of MCM2-7 protein complex is compromised in cells carrying a low basal level of FANCD2 monoubiquitination, as compared to the one present in cells with a normal level of FANCD2 monoubiquitination. Gel-filtration was done by using nuclear extracts prepared from normally growing CRL-1790 set of stable cells carrying a normal or reduced basal level of FANCD2 monoubiquitination. The endogenous FANCD2 (marked with a green frame) and all 6 members of MCM complex (a red frame) co-peak at fraction #23 (top panel), but they do not co-peak at the same fraction, but 6 members of MCM family remain co-peak together at a different fraction (#27) (bottom panel) (Fractions #9 and #30 correspond to the size markers of 2000 kDa and 669 kDa respectively).

To investigate the mechanisms by which the basal levels of monoubiquitinated FANCD2 ensure a sufficient amount of licensed-origins to fire at a normal rate (Fig. 1, 2), we performed a screen for protein functional units associated with either monoubiquitinated or un-monoubiquitinated FANCD2 in normally growing CRL-1790 cells. We found that Flag-wtFANCD2 functional units associated with a relatively higher amount of proteins sized slightly larger than 100 kDa as compared to those associated with Flag-mtFANCD2 (Fig. 3A-left panel). Mass spectrometry analysis indicated that MCM3 is an optimal, potential functional partner for Flag-wtFANCD2. We confirmed that in normally growing unstressed CRL-1790 cells, wtFANCD2, but not mtFANCD2, interacts with MCM3 by reverse immunoprecipitation (IP) and Western blotting (WB), as well as sepharose 6B chromatography (gel filtration) on the basis of their presence in each other's IP pellets and co-exiting in the same peak gel-filtration fraction, respectively (Fig. 3a-right panel and Supplementary Fig. 3A). We subsequently validated the interaction using both endogenous FANCD2 and MCM3 proteins (Fig. 3B) by reverse IP-WB analysis of pooled fractions (#21-#25) derived from the gel filtration (Fig. 3A right). We found that MCM3 is the primary MCM complex protein pulled down by FANCD2, as compared to other MCM complex members, MCM2 and MCM4 (Fig. 3B). Furthermore, we asked the nature of interaction between MCM3 and FANCD2 in terms of specific phases of cell cycle, noting that FANCD2 is only monoubiquitinated in the S-phase of normal cell cycle. We found that FANCD2 and MCM3 more efficiently associated with each other in a pool of cells enriched in S-phase as compared to the pool of cells enriched in G1 phase (Fig. 3C). Next we wanted to know how the interaction between MCM3 and FANCD2 affects the whole MCM2-7 complex, in the light of the fact that MCM3 is an essential component of the MCM complex. Surprisingly, the size distribution of MCM2-7 functional units among chromatographic fractions shows that MCM2-7 proteins and FANCD2 no longer co-peak at the same fraction of cells carrying a reduced basal level of monoubiquitinated FANCD2, as compared to corresponding control cells carrying a normal basal level of monoubiquitinated FANCD2 (Fig. 3D). Interestingly, FANCI and FANCD2 do not co-peak at the same fraction regardless cells carrying a normal or reduced basal level of FANCD2 monoubiquitination (Supplementary Fig. 3B). This is consistent with the prior observation on FANCI ChIP assay wherein FANCI did not preferentially bind to origin DNA as compared to mtFANCI, supporting our conclusion that the roles revealed here for FANCD2 is not for “ID” complex under the conditions studied. Collectively, our studies indicate that the basal level of monoubiquitinated FANCD2 (S-phase-specific) can associate with the origins as well as MCM3, and this FANCD2-mediated physical liaison between origins and MCM complexes may “tighten up” the licensed-origins, presumably, for MCM2-7 subsequently to unwind dsDNA efficiently.

### Normally monoubiquitinated FANCD2 interacts with MCM3 mainly at AA 477-480, securing enough licensed-origins ready for firing

**Figure 4:**
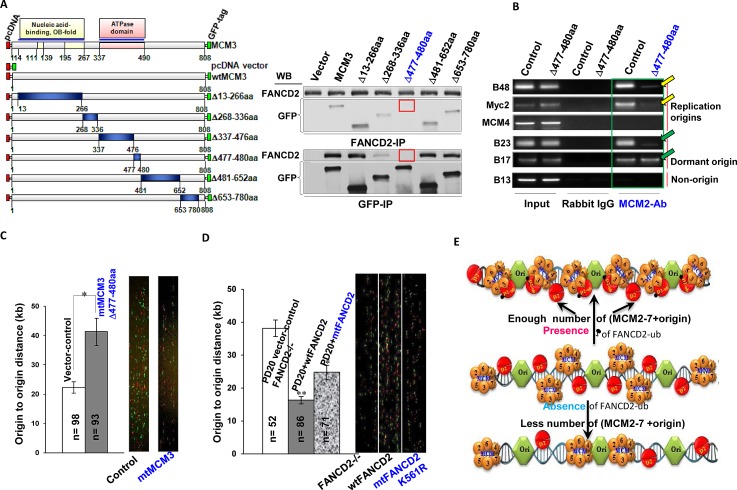
The Basal level of FANCD2 Monoubiquitination Concerts with MCM3 Mainly at 477-480AA for the Maintenance of Licensed Replication Origins to Fire at a Normal Rate A. 477-480aa of MCM3 is responsible for the interaction between MCM3 and FANCD2. Left panel: Schematic representation of a series of mutated MCM3. Right panel: GFP- MCM3 Δ477-480aa cannot interact with endogenous FANCD2. The seven sets of reverse-IP-WB were performed using CRL-1790 cells transfected with a series of deletion constructs respectively. FANCD2 and GFP- MCM3 477-480AA are not detectable in each other's IP pellets (indicated with red frames). B. MCM3 Δ477-480aa affects the number of licensed/MCMs-assembled origins. A low amount of origin DNA is associated with MCM2 proteins in CRL-1790 cells carrying MCM3 Δ477-480aa as compared to the corresponding empty vector transfected control cells. But ORC remains to interact with a similar number of origins tested. (Please see expression levels of MCM3 Δ477-480aa in CRL set of stable cells as well as ORC ChIP in Supplementary Fig. 4A, 4B, respectively). C. In CRL-1790 cell, GFP-MCM3 Δ477-480aa delays origin firing: enlarged intervals between the fired origins. In stably expressed mutant cells (CRL-1790 Δ477-480aa- Supplementary Fig. 4A) the origin density was significantly low as compared to the control cells (T test, p<0.05). Bars show standard error of the mean (SEM). D. Loss of the basal level of FANCD2 monoubiquitination leads to the enlarged intervals between the fired origins in FA cells. PD20 (FANCD2-/-) cells stably expressing wtFANCD2, K561RFANCD2 or empty vector (Supplementary Fig. 1C1) were used to perform DNA fiber assay. The origin density was significantly low in PD20+empty vector or PD20+K561RFANCD2 cells as compared to PD20+wtFANCD2 cells (T test, p<0.05). Bars show standard error of the mean (SEM). E. A working model for the normally monoubiquitinated FANCD2 to act in DNA replication initiation. In each S-phase of normal cell cycle, monoubiquitinated FANCD2 interacts with replication origins as well as the member(s) of MCM complex, such as MCM3, to provide “a temporal and spatial check” for securing an adequate number of licensed-replication origins to fire at a normal rate. Without the normal basal level of FANCD2 monoubiquitination, cells would not have an enough number of licensed origins to initiate a normal replication, thereby overtime rendering genomic instability, aging and cancer, as shown in FA.

To define the region(s) of MCM3 that are responsible for the interaction with monoubiquitinated FANCD2, we performed a series of deletion analyses on MCM3. We found that the Arginine-finger region (AA 477-480) of MCM3 is essential for the interaction between MCM3 and FANCD2 (Fig. 4A). Arginine-fingers in other MCMs play an important role in facilitating ATP hydrolysis [34], but the role of the Arginine-finger in MCM3 remains unknown. We suspect that in this case, the Arginine-finger of MCM3 may have evolved to work with normally monoubiquitinated FANCD2 to maintain the licensed-origins ready for firing. As known, a UBE or similar motifs in protein primary sequence is responsible for interacting with monoubiquitnated protein. We think that the Arginine-finger domain of MCM3 at specific temporal and spatial settings might have acted as a “3D-UBE” to interact with monoubiquitnated FANCD2. To reveal the functional significance of the interaction between MCM3 and monoubiquitinated FANCD2, we asked whether cells carrying mtMCM3 (deleted AA 477-480) show any molecular and cellular changes similar to those of cells carrying a reduced basal level of FANCD2 monoubiquitination. We generated CRL-1790 stable cell pools with or without mtMCM3 (Supplementary Fig. 4A). We found that there is a reduced amount of replication origin-containing DNA fragments associated with MCM2, but not with ORC (Fig. 4B and Supplementary Fig. 4B), as well as a low rate of origin firing (Fig. 4C) in cells carrying mtMCM3 as compared to empty vector-containing control cells. These are similar to what we observed in cells carrying compromised basal levels of FANCD2 monoubiquitination (Fig. 2B-D). In addition, we found that proliferation rates of CRL-1790 cells are similarly decreased when deficient in FANCD2 monoubiquitination at the basal level or ectopically expressing mtMCM3 (Supplementary Fig. 4C1-4). We further determined the relationship between mtMCM3 and MCM2-7 complex at the molecular level. As shown in Supplementary Figure 4D, both endogenous wtMCM3 and exogenous mtMCM3 peak at the same fraction as other MCM members, but not in the peak fraction of FANCD2. This is consistent with the results that both exogenous wt and mtMCM3 can pulldown a similar amount of other MCM members, but not FANCD2 protein (Supplementary Fig. 4E). These results suggest that mtMCM3 remains to be folded with MCM members, but not with FANCD2, leading to the loss-of-interaction of the MCM complex with FANCD2 and a resulting loss of the secure “knot” between origins and MCM complexes before firing. Further, we tested the interaction potential between FANCD2 and MCM3 in cells exposed to UV or hydroxyurea (HU) via gel filtration. We found that, unlike those shown in Figure 3D top panel, both endogenous FANCD2 and MCM3 proteins peak in different fractions (Supplementary Fig. 4F). This suggests that the optimal cooperation between FANCD2 and MCM3 (both are in the same peak fraction) is restricted in cells under normal condition. We believe that in normally growing benign cells, the basal level of FANCD2 monoubiquitination can function as a bracket to secure the bonding between the loaded-MCM complexes and replication origins, and to confer a sufficient number of licensed-origins ready for firing. In contrast, monoubiquitinated FANCD2 in stressed cells may play essential roles in signaling and repairing DNA damage or/and possibly others.

In this study, we identified an unknown role of the basal level of FANCD2 monoubiquitination in maintaining an adequate number of licensed DNA replication origins ready for firing. This is further supported by DNA fiber assay on FANCD2-null FA cells complemented with wt or mtFANCD2 cDNA, evidencing that monoubiquitination at K561 of FANCD2 is essential for proper physical intervals between the origin-firing spots on DNA fiber in normally growing cells (Fig. 4D). Our results suggest that this newly-recognized role of monoubiquitinated FANCD2 in each S-phase of normally growing cells, will give a post-licensing check on the licensed-origins before firing origins (Fig. 4E), which is distinct from its previously-known roles under conditions of stress (Supplementary Fig. 4F). Normally monoubiquitinated FANCD2, thus, plays a crucial “security” role in the maintenance of a normal origin-firing rate (Fig. 4D, E). On the contrary, a reduction in the basal level of monoubiquitinated FANCD2 leads to a low rate of replication origin firing (Fig. 2D, 4C, 4D), overtime resulting in genetic instability, aging and cancer, all of which are shown in FA.

## EXPERIMENTAL PROCEDURES

### Cell lines, antibodies, and chemicals

CRL-1970 and HEK293T cells were purchased from American Type Culture Collection (ATCC; Manassas, VA) and maintained in DMEM medium supplemented with 10% fetal bovine serum at 37 °C in 5% CO2 (v/v). Antibodies against the FANCD2, FANCL, MCM4, MCM5, MCM6 and, MCM7 were obtained from NOVUS. The anti-MCM2 and -MCM3 antibodies were from Cell Signaling. The anti-Flag and -β-actin antibodies as well as the chemicals deferoxamine (DFO), Hydroxyurea (HU), neomycin and pyrumycin were from Sigma. The anti-PCNA, -GFP and -IgG antibodies from Santa Cruz.

### ChIP assay

The ChIP assay was performed as described [35, 36]. Primer sequences used for ChIP-PCR are in Table 1. Chromatin for ChIP was prepared by fixing the cells in 1% formaldehyde for 10 min, followed by quenching with 125 mM glycine for 10 min. Briefly, fixed cells were rinsed twice with PBS and extracted in lysis buffer and diluted with RIPA ChIP buffer to give a concentration of approximately 5-6 million cells per ChIP reaction in a volume of 500 ul. Reactions were performed in 1 ml sample tubes, using 40 ul of rProtein G Agarose (Invitrogen) and 10 ug of the appropriate antibody in each ChIP reaction. Washes were performed using RIPA ChIP buffer, omitting SDS and sodium-deoxycholate, and the resulting DNA was purified using phenol/chloroform/isoamyl alcohol. Purified immunoprecipitates were dissolved in 50 ul water. Standard PCRs using 2 ul immunoprecipitated DNA were performed. PCR products were separated by electrophoresis through 1.5% agarose gels and visualized using ethidium bromide.

**Table1:** DNA primers for deletion mutation MCM3

Primer	5'-DNA sequence-3'
Δ13-266aa	TGG ACG ATG TGG AGC TGC AGA TGA GCA AGG ATG CT AGC ATC CTT GCT CAT CTG CAG CTC CAC ATC GTC CA
Δ268-336aa	CAG CCA CAT CCG TGG GTC ACG ATT TGA CTT GCT CTT C GAA GAG CAA GTC AAA TCG TGA CCC ACG GAT GTG GCT G
Δ337-476aa	ATT GCC TGT AAT GTT AAG CAG GGG GAC ATC AAT ATT CTT CTA TAG AAG AAT ATT GAT GTC CCC CTG CTT AAC ATT ACA GGC AAT
Δ477-480aa	CTA CAG GAC TCA CTG CTG TTG CTC TTC ATC ATG CTG GAT ATC CAG CAT GAT GAA GAG CAA CAG CAG TGA GTC CTG TAG
Δ481-652aa	CTG CTG TCA CGA TTT GAC TAC TTT AAG AAG GTT CTG GAG CTC CAG AAC CTT CTT AAA GTA GTC AAA TCG TGA CAG CAG
Δ653-780aa	GTT GGT CCA GTA TGC TTA CTC TTC AGT TGA GAT CCA GG CCT GGA TCT CAA CTG AAG AGT AAG CAT ACT GGA CCA AC

**Table:** 

Primer set	Sequence (5'→3')	Position in human genome (NCBI sequence accession no.)	Annealing temp (°C)
B48-F	CTCCACCCCCAAGGAAAAAG	2368081-2368062 (NT_011255.14)	60
B48-R	GGCAGGGTCCCATGCA	2368005-2368020 (NT_011255.14)	
B23-F	GTCCACCTCACTAATGCAGACAAT	33836142-33836165 (NT_ 034772.5)	60
B23-R	AGAGAAGCAGGAAGGGCTTAGAGA	33836213-33836193 (NT_ 034772.5)	
B17-F	AGCCAAGGTGCCTCTTTCAG	33833183-33833202 (NT_ 034772.5)	60
B17-R	GTCCGGGAGGAGCAGACA	33833240-33833223 (NT_ 034772.5)	
B13-F	CCCCAGGGAGTAGGTTGTGA	2363991-2363972 (NT_011255.14)	60
B13-R	TGTTATTTGAGAAAAGCCCAAAGAC	2363892-2363916 (NT_011255.14)	
Myc2-F	CTTATGCCTCTATCATTCTTCCT	1776-1795 (X00364)	56
Myc2-R	TAAATCATCGCAGGCGGAAC	1981-2000 (X00364)	
MCM4-F	AAACCAGAAGTAGGCCTCGCTCGG	12946-12969 (U63630)	58
MCM4-R	GTCTGACCTGCGGAGGTAGTTTGG	13460-13482 (U63630)	

### DNA interaction assay (Electrophoretic Mobility Shift Assays, EMSA)

Labeling of the oligonucleotides was performed with [α-32p]-dCTP by using T4 kinase enzyme (NEB). The oligonucleotide sequences used for EMSA are in Fig S1C. DNA binding reactions (21ul) contained 20mM HEPES pH 7.9, 50 mM NaCl, 20% glycerol, 2 mM EDTA, 1 mM DTT, 0.1mg/ml BSA, (~20 fmoles) radiolabelled substrate, and purified ubiquitinated or FANCD2 only protein. Reactions were incubated for 20 min at 37°C, followed by samples were loaded on a 6% non-denaturing PAGE gel run in 0.5 X TBE buffer (pH 8) and binding complexes formed gel were dried then exposed to X-ray film at −80°C O/N and developed.

### DNA fiber assay

We used the DNA fiber protocol previously developed by Sugimura et al [37] and modified by Kawabata et al., 2011 [10]. Briefly, ongoing forks were labeled with digoxigenin-dUTPs for 20 min and then with biotin-dUTPs for 30 min. Labeled cells were dropped onto slides, fixed and dipped into lysis buffer. The resulting DNA fibers were released and extended by tilting the slides. Incorporated dUTPs were then visualized by immunofluorescent detection using anti digoxigenin-Rhodamine (Roche) and streptavidin-Alexa-Fluor-488 (Invitrogen).

### Chromatin Immunofluorescence Staining

CRL-1970 stable cells (1 x 10^3^) were seeded onto sterilized black well slides, and were pre-extracted with phosphate-buffered saline (PBS) containing 0.3% Triton X-100 for 5 min and then fixed with pre-chilled 3.7% paraformaldehyde/PBS for 15 min at 4 °C and quenching with 125mM of glycine for 5-10 min at room temperature. Next, cells were permeabilized with 0.2% pre-chilled Triton-X 100/PBS for 5 min at 4 °C, blocked with 3% goat serum/PBS for 30 min at RT, and subsequently incubated with appropriate antibody at 4°C overnight. After washing, cells were further incubated with appropriate Alexa Fluor secondary antibody at 1:1000 dilutions for 30 min at room temperature. After incubation with secondary antibody, cells were washed and mounted in Vectashield mounting media with DAPI (Vector Laboratories, Burlingame, CA). Cells were observed with a 40X objective lens on a Olympus BX-51microscope equipped with a SenSys fluorescence CCD camera. Images were acquired and analyzed using MetaMorph version 4.5 Premier.

### Gel filtration

Cytoplasmic and nuclear fractions were prepared essentially as described in protocols provided by the manufacturer (Pierce) and as we did before [22]. The NE-PER Nuclear and Cytoplasmic Extraction Reagents Kit (Pierce) was used. The nuclear extract was directly applied to a Superose 6B column equilibrated with the column running buffer containing 20mM HEPES (pH 7.9), 200mM NaCl, 1mM dithiothreitol (DTT), 0.1mM phenylmethylsulfonyl fluoride (PMSF), 5 mg/ml leupeptin, 2 mg/ml aprotinin, 0.1% NP-40 and 5% glycerol. Fractions were collected and analyzed by SDS–PAGE and immunoblotting. Dextra blue and were used to determine the sizes of fractions

### Immunoprecipitation and Immunoblotting

Essentially as described previously [19, 22, 36, 38], the whole cell lysates or nuclear extracts, prepared from cells transfected with appropriate plasmid constructs, and cells with empty vectors, used as the experimental controls accordingly, were mixed with 1 μg Flag antibody or GFP antibody, and 40 μl of flurry IgA beads (Invitrogen) for rotating overnight at 4°C. The immunoprecipitates were washed four times with the IP buffer (20mM HEPES (pH 7.9), 350mM NaCl, 0.1% NP-40, 1mM DTT, 0.2mM PMSF, 2mg/ml leupeptin and 2 mg/ml aprotinin). The eluate from IP-pellets was Western blotted for appropriate antibodies. Control antibody rabbit IgG was used simultaneously when IPs was performed. For immunoblotting, briefly, the transfer-ready membrane was blocked overnight in TBS (10 mM Tris-HCl [pH 7.5], 150 mM NaCl) containing 5% nonfat milk and 0.1% Tween-20 at 4°C, followed by incubation with appropriate primary antibody. The secondary antibodies were horseradish peroxidase-conjugated anti-mouse, anti-rabbit, and anti-goat antibodies were used at a 1:10000 dilution. Actin was used as a protein loading control.

### Mass spectroscopy

Flag-wtFANCD2 and -mtFANCD2 was separated by 8-16% gradient SDS–PAGE and stained with Coomassie blue. Protein bands were excised, digested with tryspin and analysed by MS/MS mass spectrometry for peptide fragmentation at the Harvard Medical School Taplin Biological Mass Spectrometry Facility.

### Cell Fractionation

We used the cell fractionation method originally described in Andersen et al., 2002 and as we did before[18]. Briefly, cells were washed three times with ice cold PBS, resuspended in 5 ml of buffer A (10 mm HEPES-KOH (pH 7.9), 1.5 mM MgCl_2_, 10 mM KCl, 0.5 mM DTT), and Dounce homogenized 10 times using a tight pestle. Dounce homogenized nuclei were centrifuged at 228 × *g* for 5 min at 4 °C. The supernatant represents the cytoplasmic fraction. The nuclear pellet was resuspended in 3 ml of 0.25 mM sucrose, 10 mM MgCl_2_; layered over 3 ml of 0.35 mM sucrose, 0.5 mM MgCl_2_; and centrifuged at 1,430 × *g* for 5 min at 4 °C. The clean, pelleted nuclei were resuspended in 3 ml of 0.35 mM sucrose, 0.5 mM MgCl_2_ and sonicated for 6 × 10 sec. The sonication was checked using phase-contrast microscopy, ensuring that there were no intact cells and that the nucleoli were readily observed as dense, refractile bodies. The sonicated sample was then layered over 3 ml of 0.88 mM sucrose, 0.5 mM MgCl_2_ and centrifuged at 2,800 × *g* for 10 min at 4 °C. The pellet contained the nucleoli, whereas the supernatant consisted of the nucleoplasmic fraction. The nucleoli (chromatin) were then washed by resuspension in 500 μl of 0.35 mM sucrose, 0.5 mM MgCl_2_ followed by centrifugation at 2,000 × *g* for 2 min at 4 °C. This supernatant contained chromatin fraction and anti-histone H4 was used as protein loading control.

### Gene knockdown by Lentivirus ShRNA

For lentiviral transduction essentially as described previously [39]. A set of five pLKO.1 plasmids containing shRNA targeting FANCL (purchased from Thermo scientific, Open BioSystems) along with pLKO.1 empty vector were used to generate corresponding lentiviruses. CRL-1970 and HEK293 cells were then infected with these viruses according to the protocol provided. Infected cells were pool-selected with puromycin 48 hours post-infection, and FANCL knock down was verified using FANCL antibody (NOVUS).

### Site-directed mutagenesis and deletion constructs

A full-length human MCM3 were generated by PCR amplification using pcDNA3.1/NT-GFP-TOPO TA Expression Kits (Invitrogen) with primes (5'- ATG GCG GGT ACC GTG GTG) and (5'- TCA GAT GAG GAA GAT GAT GCC C). Deletion mutants of MCM3s were generated by PCR amplification using template pcDNA3.1/NT-GFP-TOPO MCM3 plasmid, as above. The deletions-mutations were generated using QuikChange Site-Directed Mutagenesis Kit (Stratagene). The sequence of primers sets in Table 1. Plasmids were purified using Qiagen plasmid purification kits (QIAGEN Inc.). One of deletion mutation construction (Δ337-476aa) might be lethal, we were failed to get bacterial colonies.

## SUPPLEMENTARY FIGURES


