# Supplementary material for: Basal level of FANCD2 monoubiquitination is required for the maintenance of a sufficient number of licensed-replication origins to fire at a normal rate
Source: Oncotarget. 2014 Mar 13;5(5):1326–37. doi: 10.18632/oncotarget.1796 (PMC4012723; doi:10.18632/oncotarget.1796)
Supplement: Supplementary file 1 [file oncotarget-05-1326-s001.pdf]

Supplementary Figure 1

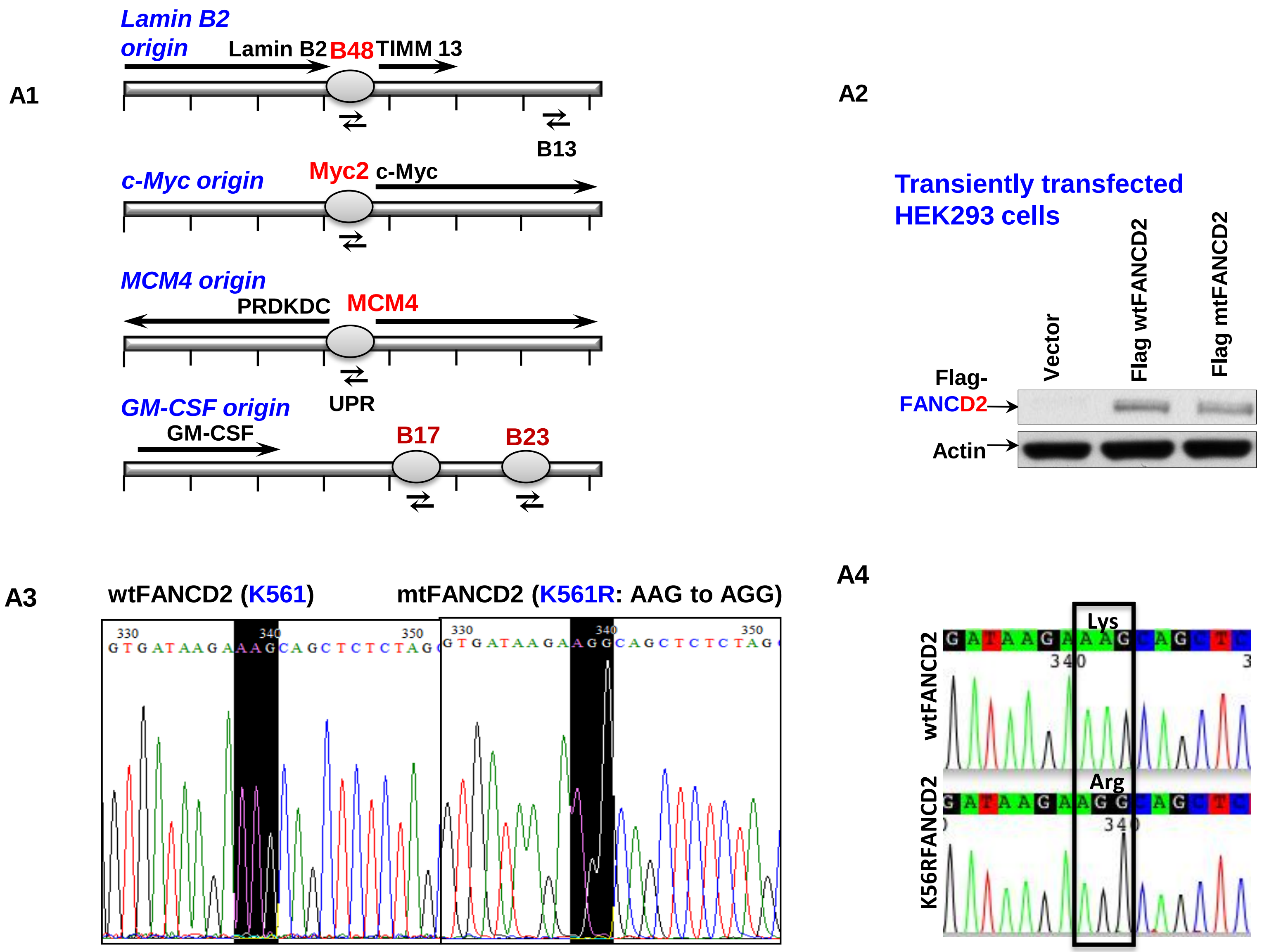

Supplementary Figure 1

B1

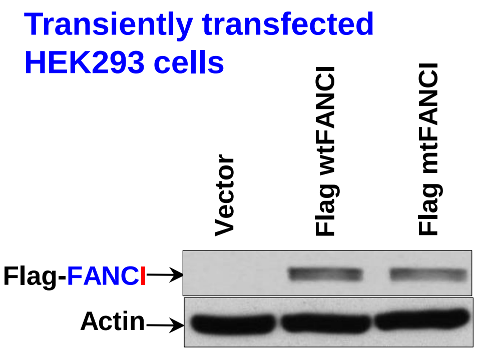

B2

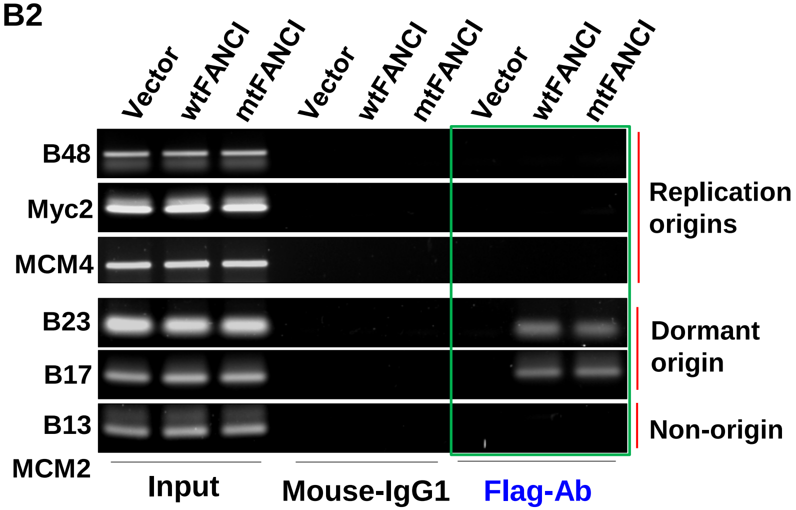

Supplementary Figure 1

C1

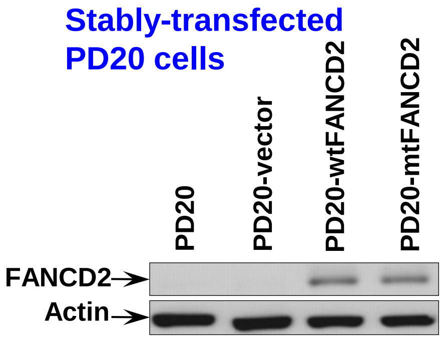

C2

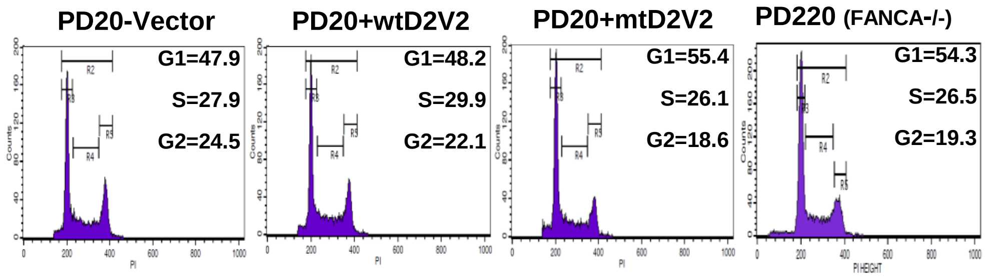

C3

Comparable FANCD2 protein levels

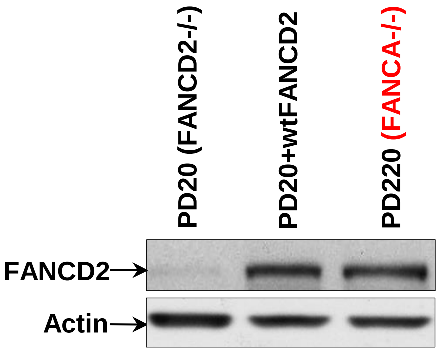

D

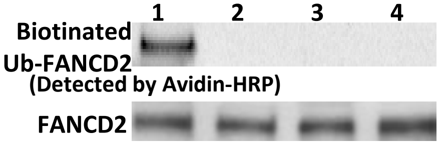

1. Wt FANCD2 in the ub reaction
2. mtFANCD2 in the ub reaction
3. wtFANCD2
4. mtFANCD2

Biotinated-Ub-FANCD2 indicates the monoubiquitnated FANCD2
Ub-ubiquitin

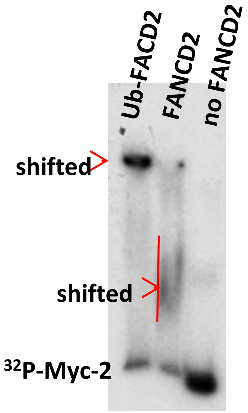

Supplementary Figure 2

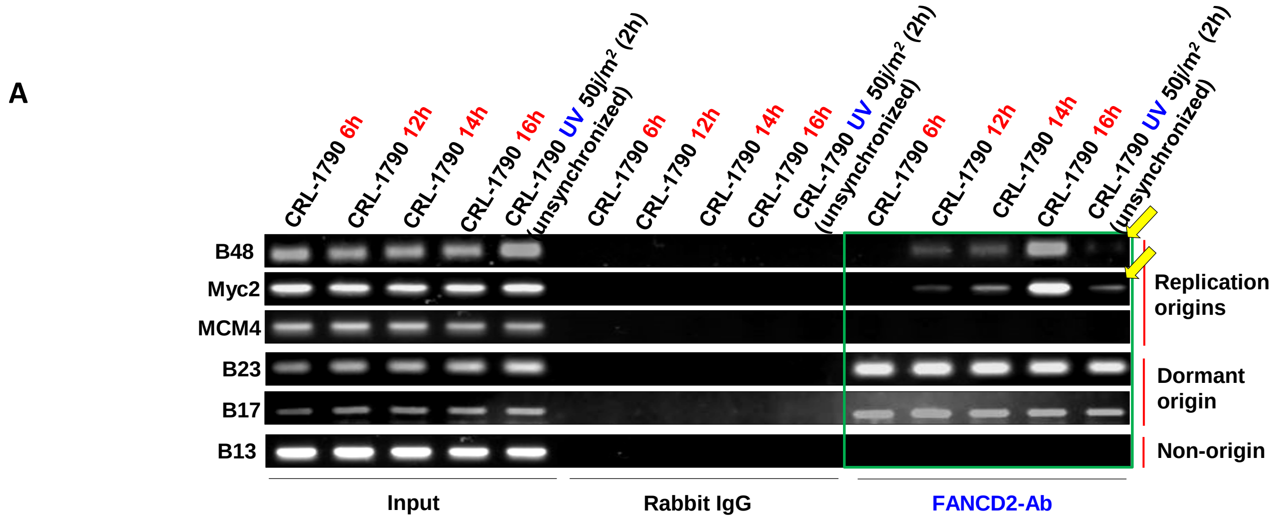

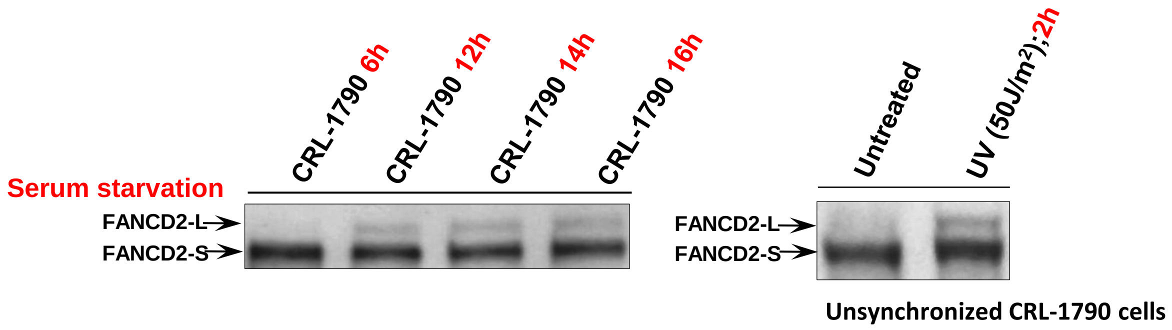

Time 0: The end of Synchronization by Starvation
FANCD2-L: Monoubiquitinated FANCD2
FANCD2-S: Un-monoubiquitinated FANCD2

Supplementary Figure 2

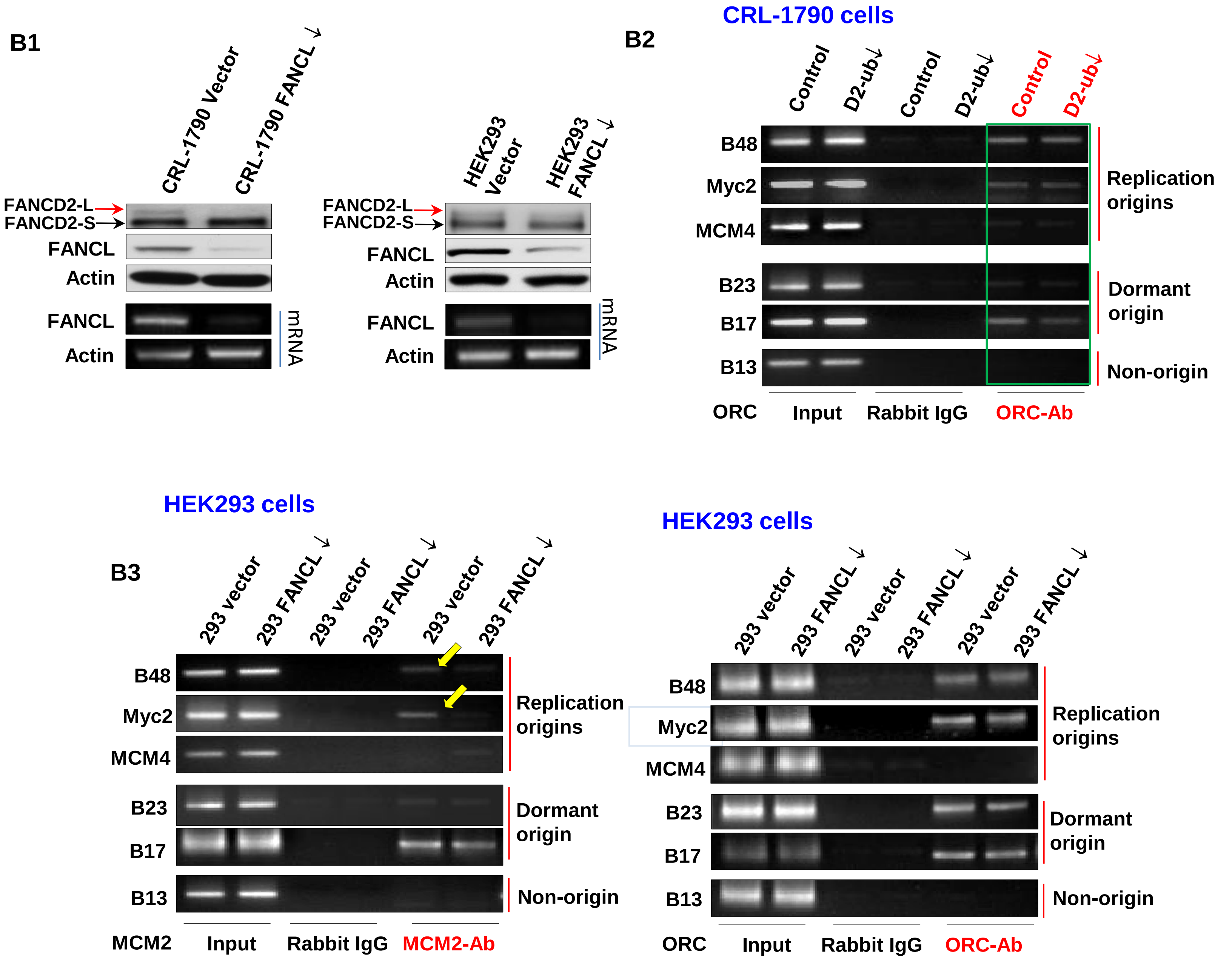

Supplementary Figure 2

B4

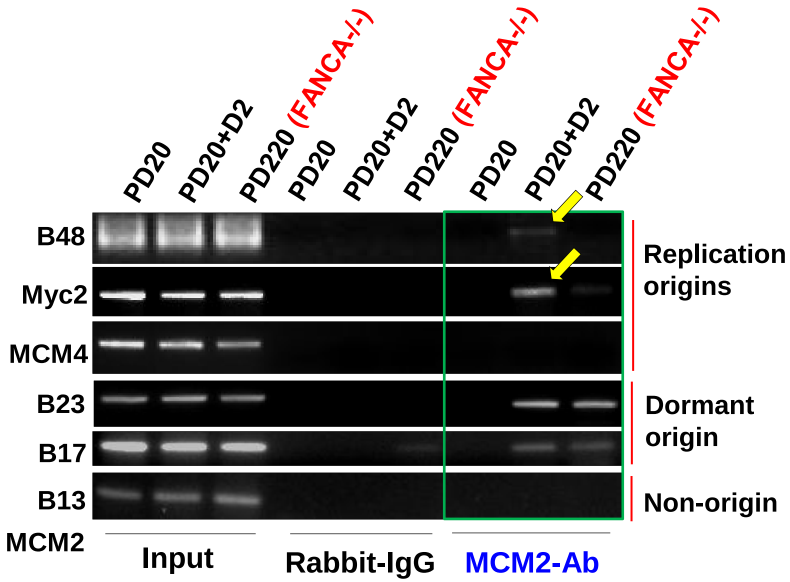

C1

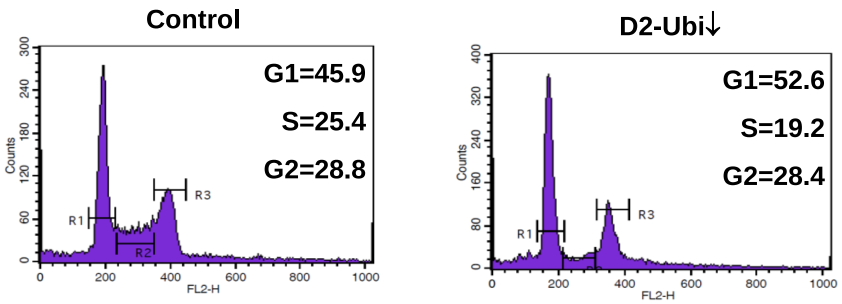

C2

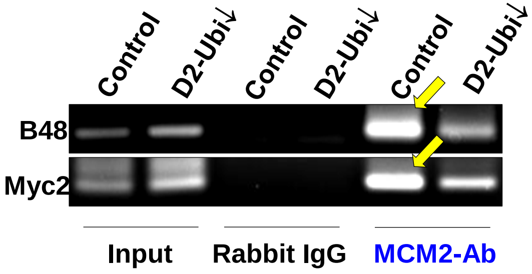

Supplementary Figure 2

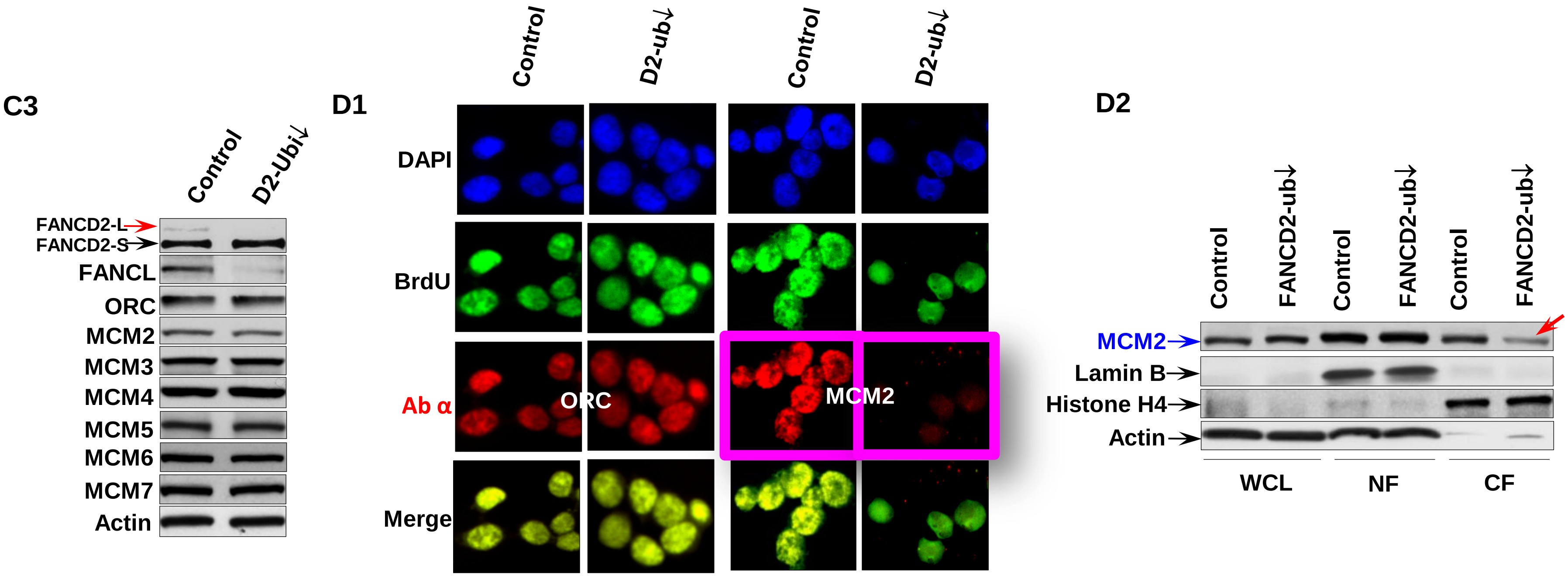

Supplementary Figure 3

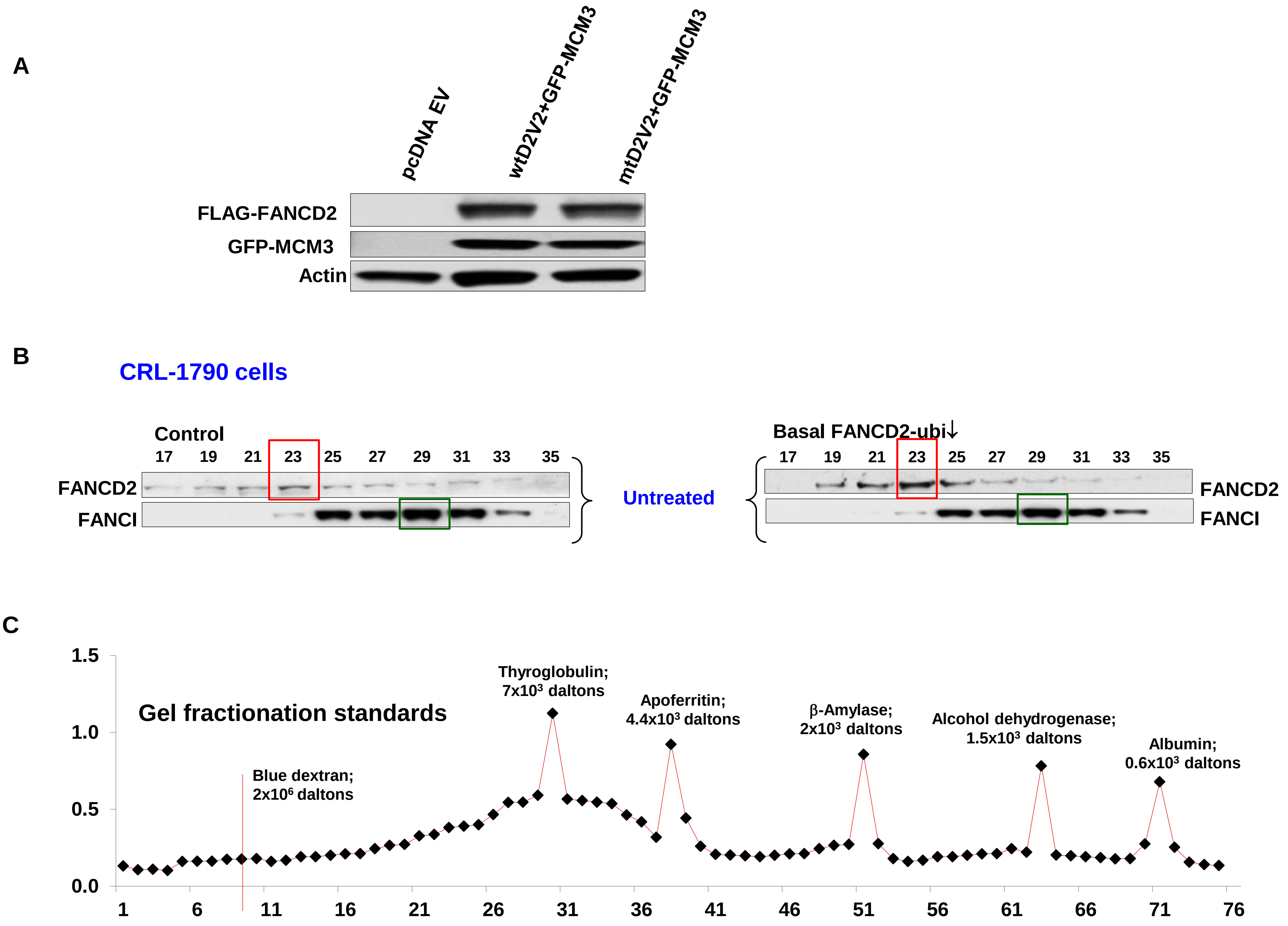

Supplementary Figure 4

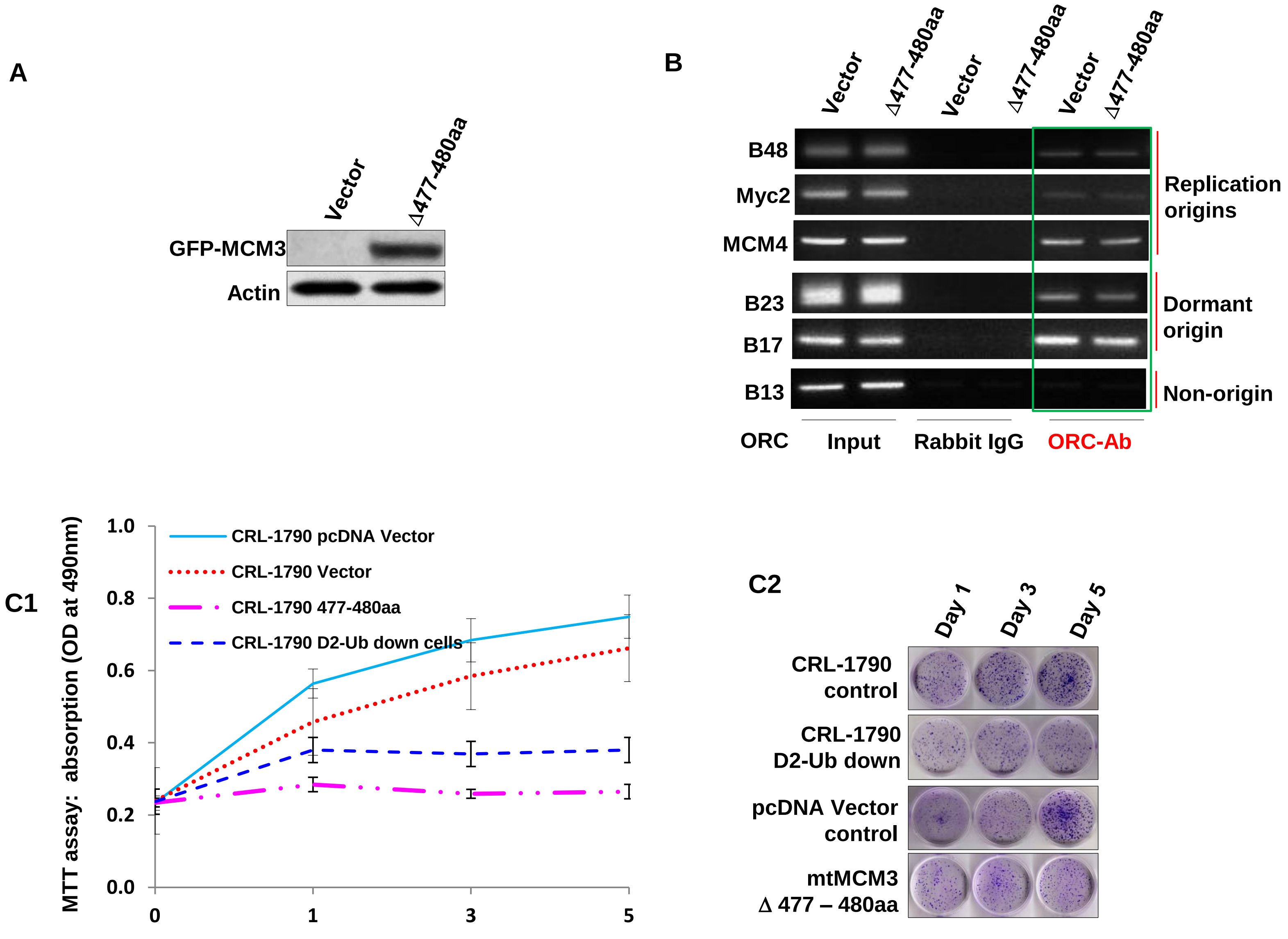

Supplementary Figure 4

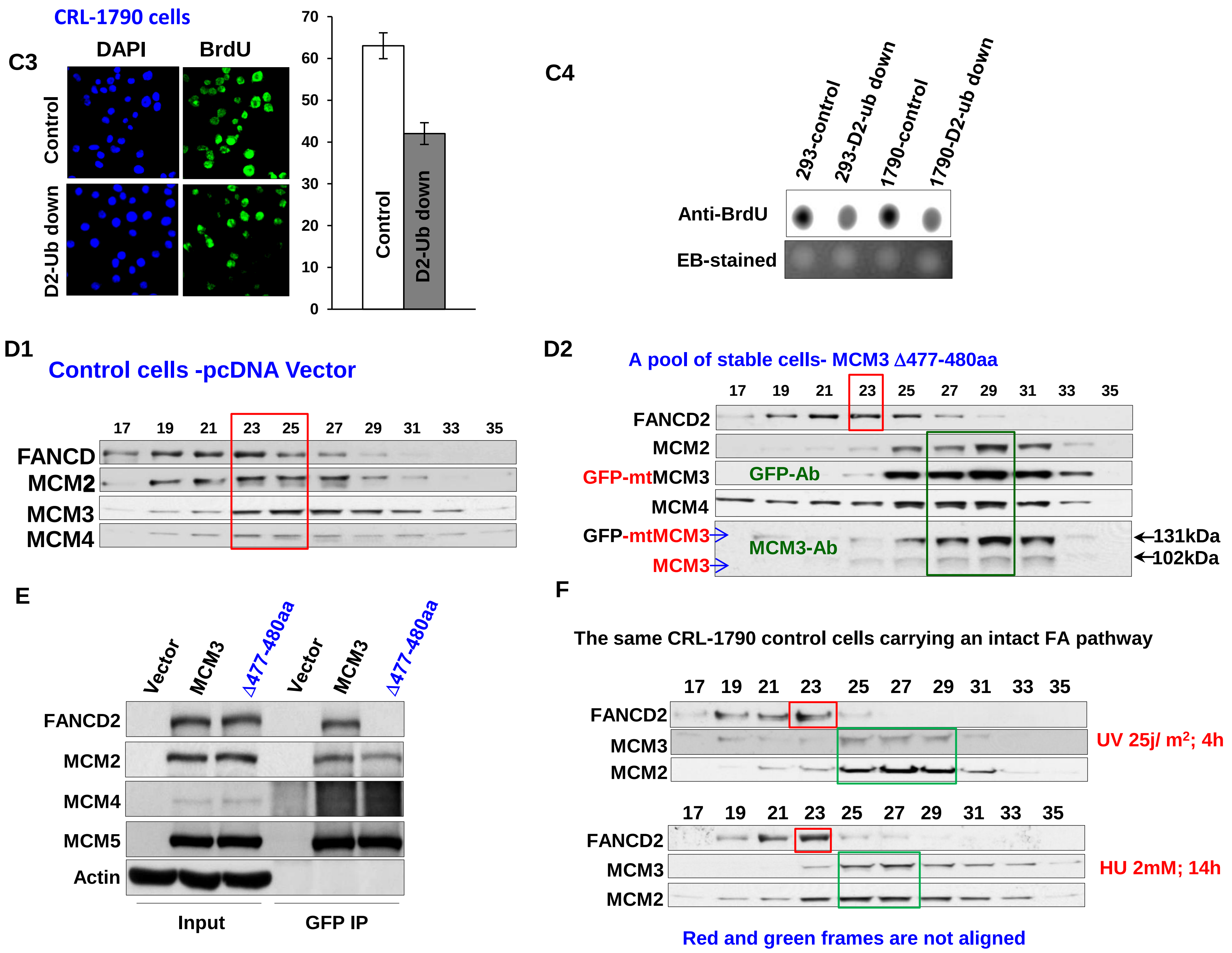

Legends for Supplementary figures

Figure S1.

SA1. The information of origins used

SA2. Equal amount of FANCD2 protein is expressed in transiently transfected HEK293 cells for the ChIP assay shown in Figure 1A.

SA3,4 Sequencing confirmation of SF-Flag- wtFANCD2 and SF-Flag-mtFANCD2 K561R

SB1. Equal amount of FANCI protein is expressed in transiently transfected HEK293 cells.

SB2. Neither wtFANCI nor mtFANCI K523R shows the capability to preferentially interact with replication origins tested.

SC1. Equal amount of FANCD2 protein is expressed in stably-transfected PD20 FA cells (FANCD2-/-).

SC2. PD220 FA cells and PD20 derivative cells, carrying empty vector, wtFANCD2, or mtFANCD2 K561R, show a similar cell cycle profile with an exception of 2-3% less in the population of S phase cells when presence of mtFANCD2 or inactivated FANCD2, as compared to wtFANCD2.

SC3. The level of FANCD2 proteins shown in PD20+FANCD2 cells is comparable to that in PD220 FA cells (FANCA-/-).

SD. Left: An equal amount of FANCD2 proteins used in the assay.

Right: in vitro binding assay was repeated and Myc-2 binding to monoubiquitinated FANCD2 (ub-D2) was shown.

Figure S2.

S2A. CRL-1790 cells were synchronized by starvation for 60 hrs. Cells cultured in completed medium for 6, 12, 14, 16 hrs were collected for conducting FANCD2-ChIP or WB. Unsynchronized CRL-1790 cells treated with or without UV were also collected for corresponding assays.

ChIP results show that the level of monoubiquitinated FANCD2 is positively correlated with the amount of B48 or Myc2-containing DNA segments pulled down by FANCD2 antibodies in normally growing synchronized cells. But, regardless of the level of monoubiquitinated FANCD2 in UV treated cells, FANCD2 antibodies barely pulled down B48 or Myc2 origin-containing DNA segments.

S2B1. Two sets of stable cell pools carry different levels of FANCL expression, and thus different basal levels of FANCD2 monoubiquitination. As indicated, FANCD2-L (long form, monoubiquitinated) is decreased in FANCL-down-regulated cells (FANCD2-S, un-monoubiquitinated).

S2B2. ORC antibodies, unlike MCM2 antibodies, can pulldown a similar amount of origin-containing DNA fragments in CRL-1790 cells with or without changed basal level of FANCD2 monoubiquitination.

S2B3. What shown in Figures 2B & S2B1 was reproducible in a different cell system (human HEK 293 cells).

S2B4. MCM2 ChIP was performed in FA derivative cells. MCM2 antibodies pulled down a less amount of B48 or Myc2-containing DNA segments in cells carrying inactivated FANCD2 (PD220 FA cells: FANCA-/-), as compared to the PD20 FA cells carrying wtFANCD2.

S2**C.** MCM2 remains to show the low capacity to interact with replication origins tested upon the adjusted level of ChIP input for an equal amount of S phase chromatin.

S2C1. Cell cycle profiles of CRL-1790 set of stable cell Paris isogenic to the basal level of FANCD2 monoubiquitination (Figure S2B1 right panel). Cells carrying a decreased basal level of FANCD2 monoubiquitination show a low percentage of S phase cells 19.2% versus 25.4% in control cells.

S2C2. MCM2 ChIP was performed by adding more input of ChIP lysate to reach a similar amount of S phase chromatin for both control and D2-ub down cells. MCM2 antibodies remain to be able to pull down much more origin-containing DNA fragments as revealed in Figure 2B.

S2C3. FANCL knockdown does not affect the expression levels of ORC and MCM2-7 proteins in CRL-1790 set of stable cells (Figure 2A1) or HEK293 cells of stable cells (not shown).

S2D1. The chromatin level of MCM2 protein, but not ORC, is decreased in S phase cells carrying a compromised basal level of FANCD2 monoubiquitination (D2-ub↓) (Green color of BrdU staining is an indicative of S phase cells).

S2D2. Western blotting shows the chromatin level of MCM2 protein is decreased in cells carrying a reduced basal level of FANCD2 monoubiquitination (FANCD2-ub down) as compared to the control cells carrying a normal basal level of FANCD2 monoubiquitination. Lamin B and Histone 4 were used as nuclear plasma (NF) and chromatin fraction (CF) controls respectively.

Figure S3

S3A. Transfection efficiency is equal in cells transfected with wt or mtFANCD2. The resulting lysates were used for Flag-IP (Figure 3A-left) for Mass spec analysis.

S3B. FANCI does not co-peak with FANCD2 in the fraction (#23) where MCM2-7 levels are the highest.

S3C. The panel of makers was run through gel-filtration column to assign the relative sizes to protein complexes in each fraction.

Figure S4

S4A. The establishment of stable pool cells, which express GFP- MCM3 Δ477-480aa at null or a higher level.

S4B. ORC interacts equally with origin DNA fragments in cells with or without GFP-MCM3 Δ477-480aa.

S4C1. CRL-1790 cells with silenced FANCL or mtMCM3 show a similar low proliferation rate compared to the control cells.

S4C2. The cell growth images of both sets of cells indicate the cells either with down-regulated FANCD2 monoubiquitination or mtMCM3 have a similarly low growth rate.

S4C3. BrdU-labeling *in situ* images indicate a low growth rate for CRL-1790 cells carrying a reduced basal level of FANCD2 monoubiquitination, as compared to the control cells (the same for 293 stable cell pairs not shown) (two sets of stable cells shown in Figure S2B1). (Bars show the percentages of cells labeled with BrdU) (10 fields were counted to make the bar graph).

S4C4. The same batch of cells was used to isolate the genomic DNA. 50ng of sheared genomic DNA was loaded on the nylon membrane (EB staining to show the equal loading), which was subsequently cross-linked and detected with anti-BrdU antibodies. The strong BrdU signaling is observed in control cells as compared to the cells carrying a compromised basal level of monoubiquitinated FANCD2, indicating the cells carrying a reduced basal level of FANCD2 monoubiquitination have a slow growth rate.

S4D. The stable cell pairs (Figure S3A) carrying empty V control or GFP-tagged mtMCM3 were prepared for gel filtration study. In control cells, MCMs and FANCD2 co-peak at Fraction

#23 (S4D1). However, In mtMCM3-expressing cells, mtMCM3 and MCMs remain to co-peak in the same fraction #27 but not with FANCD2 (S4D2).

S4E. Empty vector, GFP-wtMCM3 and GFP-mtMCM3 were transiently transfected into CRL-1790 cells, respectively. Subsequently, GFP IP-WB was performed using lysates isolated from these transfected cells 48 hr post-transfection. Anti-MCM2, 3, or 5 antibodies were used to detect the IP elutes. All MCM members detected were shown to equally associate with GFP-wtMCM3 or GFP-mt MCM3.

S4F. Unlike what shown in non-stressed CRL-1790 cells (Figure 3D-top panel), FANCD2 is no longer co-peaked with MCM3/MCMs in CRL-1790 cells upon UV or HU treatment. Fractions #9 and #30 correspond to the size marker of 2MkDa and 679kDa respectively.
